# Ballet Rehabilitation: A Novel Return to Sport Protocol

**DOI:** 10.7759/cureus.27896

**Published:** 2022-08-11

**Authors:** Laurie Glasser, Marie Frey, Giulia C Frias, Bobby Varghese, Justin X Melendez, Joseph D Hawes, Jared Escobar, Brian M Katt

**Affiliations:** 1 Department of Orthopaedic Surgery, Jersey Shore University Medical Center, Neptune, USA; 2 Physical Therapy, Ivy Rehabilitation, Toms River, USA; 3 Department of Orthopaedic Surgery, Robert Wood Johnson Medical School, New Brunswick, USA; 4 Department of Surgery, University of Texas Southwestern Medical Center, Dallas, USA

**Keywords:** dance, ballet, ballet reinjury, ballet injury, ballet rehabilitation, dance rehabilitation, return to play, sports rehabilitation, return to sport, rehabilitation protocol

## Abstract

Dance injuries and re-injuries are common but can be difficult to rehabilitate because of the unique demands and motor skills required. During tissue healing, pain resolves prior to tissue maturation and re-injury often occurs if the original injury is not properly rehabilitated.

The purpose of this narrative review is to analyze the existing literature addressing ballet injury, re-injury, and recovery, and to provide clinicians with timing guidelines for entering and implementing a Return to Sport (RTS) ballet rehabilitation protocol designed to prevent re-injury by progressive, sport-specific tissue loading. Thus far, a literature-based ballet-specific and body region-specific late-stage rehabilitation RTS protocol has not been established. The authors sought to address this literature gap by combining this comprehensive narrative review with our extensive clinical expertise to develop a late-stage rehabilitation RTS protocol to help guide medical clinicians treating injured ballet dancers.

## Introduction and background

Healing and rehabilitation of ballet injuries

One of the many challenges in all of sports medicine is determining when to initiate a return to sport (RTS) protocol, and exactly how to safely progress an athlete back to full participation without re-injury. The purpose of this review is to fill a void in the literature by addressing how and when a medical dance professional (physician or other licensed medical provider) should begin and safely progress a dancer through a ballet-specific RTS protocol. There is a scarcity of information available to assist medical professionals in guiding an injured ballet dancer back toward full participation during late rehabilitation while avoiding re-injury and chronic musculoskeletal issues.

After reviewing the literature, we defined objective criteria to assess a ballet dancer’s readiness to RTS. As set forth in this review, once an injured ballet dancer meets defined criteria, they can qualify to enter a functional RTS phase with our “Return to Ballet Protocol” (Table 1). We found no ballet-specific, body region-specific late-stage rehabilitation protocols; therefore, we developed a protocol to assist medical providers in progressing injured ballet dancers to full participation during the functional (late-stage rehabilitation) RTS phase (Table 1) [1-21].

**Table 1:** Return to Ballet Protocol ROM: range of motion; ACL: anterior cruciate ligament; MCL: medial collateral ligament; MPFL: medial patellofemoral ligament; DJD: degenerative joint disease [1-21].

Foot and Ankle Injury Return to Ballet Protocol (e.g., midtarsal joint sprain, ankle sprain, Achilles tendinitis, anterior ankle impingement, posterior ankle impingement, stress fracture, plantar fasciitis)	
Stage 1	For any single leg skills, the injured leg is the working leg. No restrictions on the supporting leg except no fondu. Begin this stage in a sneaker or in a jazz shoe but master all steps without support prior to moving to the next stage. All steps should be completed with barre support first progressing from two-handed support to one-handed support. Progress to center when pain-free. Demi plié limited to half ROM, tendu from 1^st^ position progressing to 5^th^ position when pain-free, rond de jambe a terre, relevé on two feet.
Stage 2	For any single leg skills, the injured leg is the working leg. No restrictions on the supporting leg. When the injured leg is the working leg, the dancer is cleared for pirouettes, attitude turns, fouetté turns, a la seconde turns, and float turns. No turns with the injured leg as the supporting leg. All steps should be completed with barre support first progressing from two-handed support to one-handed support. Progress to center when pain-free. All steps in stage 1 plus: full demi plié progressing to grande plié as tolerated, degagé, frappé on relevé so injured leg does not strike floor, echappé, relevé on one foot, bourrée, pas de bourrée, grande battement, developpé, arabesque, penché.
Stage 3	All steps in stage 2 plus: full barre without restriction, fondu with injured leg as supporting leg, non-modified frappé, detourné, chainé turns, piqué turns, pirouette with injured leg as supporting leg beginning with singles and progressing until previous level is reached (no restriction with pirouette when non-injured leg is supporting leg), attitude turns with injured leg as supporting leg (no restriction with pirouette when non-injured leg is supporting leg).
Stage 4	All steps in stage 3 plus: fouetté turns with injured leg as supporting leg, a la seconde turns with injured leg as supporting leg, float turns with injured leg as supporting leg, glissade, chassé, pas de chat, soubresaut, changement (no beats).
Stage 5	All steps in stage 4 plus: sissonne, assemblé, jumps with beats, all petit allégro except temps levé.
Stage 6	All steps in stage 5 plus: grande jeté, tour jeté, saut de chat, temps levé. Return to partner work. Pointe work can be started after pain-free completion of this stage.

Knee Injury Return to Ballet Protocol (e.g., patellofemoral syndrome, patellar tendinitis, quad tendinitis/osis, Osgood Schlatter, ACL tear, MCL sprain or tear, MPFL tear, meniscal tear, posterior capsulitis)	
Stage 1	For any single leg skills, the injured leg is the working leg. No restrictions on the supporting leg except no fondu. All steps should be completed with barre support first progressing from two-handed support to one-handed support. Progress to center when pain free. Tendu, rond de jambe terre, grande battement, développé, arabesque, penché, bourrée, pas de bourrée, demi plié in first and second position, grande plié in second position, degagé, frappé, echappé, relevé in first and second position.
Stage 2	When the injured leg is the working leg, the dancer is cleared for pirouettes, a la seconde turns, and float turns. No turns with injured leg as supporting leg. All steps in stage 1 plus: demi and grande plié in all positions, relevé in all positions, detourné, rond de jambe en l’air at 45 degrees progressing to 90 degrees then dancer’s end range with the injured leg as the working leg. No restriction on the supporting leg (unless pain).
Stage 3	When the injured leg is the working leg, the dancer is cleared for turns from stage 2 plus attitude turns and fouetté turns. All steps in stage 2 plus: full barre without restriction, include fondu with injured leg as supporting, sissonne landing on uninjured leg, chainé turns, pirouettes with injured leg as supporting leg beginning with singles and progressing until previous level is reached (no restriction with pirouette when non-injured leg is supporting leg), attitude turns.
Stage 4	All steps in stage 3 plus: piqué turns, fouetté turns, a la seconde turns, float turns, soubresaut, changement, pas de chat, glissade, chassé.
Stage 5	All steps in stage 4 plus: assemblé, temps levé, all petit allégro
Stage 6	All steps in stage 5 plus: saut de chat, sissonne landing on injured leg, grande jeté, tour jeté. Return to partner work. Pointe work can be started after pain-free completion of this stage.

Anterior Hip Injury Return to Ballet Protocol (e.g., snapping hip, hip flexor strain or tendinitis, bony avulsions, impingement, sartorius enthesopathy, hip labral tear, hip DJD, femoral neck stress fracture)	
Stage 1	All steps should be completed with barre support first progressing from two-handed support to one-handed support. Progress to center when pain-free. Relevé, detourné, demi plié in all positions, bourrée, pas de bourrée, echappé, fondu.
Stage 2	When the injured leg is the supporting leg, the dancer is cleared for pirouettes, attitude turns, fouetté turns, a la seconde turns, and float turns. No turns with injured leg as working leg. All steps in stage 1 plus: rond de jambe a terre, grande pliés in all positions, chainé turns, piqué turns, glissade, chassé, passé, frappé, degagé.
Stage 3	All steps in stage 2 plus: pirouettes with injured leg as working leg, pas de chat, temps levé, assemblé, sissonne, rond de jambe en l’air at 45 degrees, penché with injured leg as the working leg, arabesque, changement, soubresaut.
Stage 4	All steps in stage 3 plus: grande battement to 90 degrees with the injured leg as the working leg (no restriction on the supporting leg), développé to 90 degrees with the injured leg as the working leg (no restriction on the supporting leg), rond de jambe en l’air at 90 degrees with the injured leg as the working leg (no restriction on the supporting leg), penché with injured leg as the support leg.
Stage 5	All steps in stage 4 plus: grande battement at dancer’s end range, développé at dancer’s end range, rond de jambe en l’air at dancer’s end range, attitude turns, fouetté turns, a la seconde turns, float turns.
Stage 6	All steps in stage 5 plus: saut de chat, grande jeté, tour jeté. Return to partner work. Pointe work can be started after pain-free completion of this stage.

Posterior Hip Injury Return to Ballet Protocol (e.g., piriformis or obturator internus strain)	
Stage 1	All steps should be completed with barre support first progressing from two-handed support to one-handed support. Progress to center when pain-free. Limit turnout to pain-free range relevé, demi plié in first and second positions, grande plié in first and second positions, fondu from first position, bourrée, pas de bourrée, echappé, passé, rond de jambe a terre, chainé turns, chassé.
Stage 2	When the injured leg is the supporting leg, the dancer is cleared for pirouettes, attitude turns, fouetté turns, a la seconde turns, and float turns. No turns with injured leg as working leg. All steps in stage 1 plus: detourné, demi plié in all positions, grande plié in all positions, fondu from 5^th^ position, frappé, degagé, piqué turns.
Stage 3	All steps in stage 2 plus: grande battement, développé, pirouettes with injured leg as working leg, glissade, pas de chat, assemblé, sissonne, changement, soubresaut.
Stage 4	All steps in stage 3 plus: fouetté turns with injured leg as working leg, a la seconde turns with injured leg as working leg, float turns with injured leg as working leg, penché with injured leg as support leg.
Stage 5	All steps in stage 4 plus: rond de jambe en l’air, attitude turns, penché with injured leg as working leg, arabesque.
Stage 6	All steps in stage 5 plus: saut de chat, grand jeté, tour jeté. Return to partner work. Pointe work can be started after pain-free completion of this stage.

Hamstring Injury Return to Ballet Protocol	
Stage 1	All steps should be completed with barre support first progressing from two-handed support to one-handed support. Progress to center when pain-free. Relevé, detourné, demi plié, grande plié, fondu, bourrée, pas de bourrée, rond de jambe a terre, passé, chainé turns, pirouettes.
Stage 2	All steps in stage 1 plus: soubresaut, changement without beats, piqué turns, echappé, degagé, chassé, pas de chat, soubresaut.
Stage 3	All steps in stage 2 plus: changement with beats, rond de jambe en l’air at 45 degrees, développé to 90 degrees, arabesque to 90 degrees, a la seconde turns, float turns.
Stage 4	All steps in stage 3 plus: développé to dancer’s end range, battement, penché at half of normal extension, assemblé, sissonne, rond de jambe en l’air to 90 degrees, temps levé.
Stage 5	All steps in stage 4 plus: rond de jambe en l’air to dancer’s end range, full extension penché.
Stage 6	All steps in stage 5 plus: saut de chat, grande jeté, tour jeté. Return to partner work. Pointe work can be started after pain-free completion of this stage.

Back-Extension Injury Return to Ballet Protocol	
Stage 1	All steps should be completed with barre support first progressing from two-handed support to one-handed support. Progress to center when pain-free. All pliés, detourné, tendu, rond de jambe a terre, degagé, frappé, fondu, echappé, battement devant and to the side, développé devant and to the side, cambré to the front, attitude devant, bourrée, pas de bourrée.
Stage 2	All steps in stage 1 plus: cambré to the side, pirouettes, chainé turns, piqué turns, fouetté turns, a la seconde turns, float turns.
Stage 3	All steps in stage 2 plus: arabesque to 45 degrees, chassé, glissade, pas de chat.
Stage 4	All steps in stage 3 plus: penché through 50% ROM, arabesque to 90 degrees, battement derierre to 90 degrees, attitude derierre, rond de jambe en l’air.
Stage 5	All steps in stage 4 plus: cambré to the back, penché through full range, arabesque to full range, attitude turns, soubresaut, changement, sissonne, assemblé, temps levé.
Stage 6	All steps in stage 5 plus: saut de chat, grande jeté, tour jeté. Return to partner work. Pointe work can be started after pain-free completion of this stage.

Back-Flexion Injury Return to Ballet Protocol	
Stage 1	All steps should be completed with barre support first progressing from two-handed support to one-handed support. Progress to center when pain-free. All pliés, detourné, tendu, rond de jambe a terre, degagé, frappé, fondu, echappé, battement derierre and to the side, développé derierre and to the side, cambré to the back, attitude derrière, bourrée, pas de bourrée.
Stage 2	All steps in stage 1 plus: cambré to the side, pirouettes, chainé turns, piqué turns, fouetté turns, a la seconde e turns, float turns.
Stage 3	All steps in stage 2 plus: attitude devant, piqué turns, attitude turns, chassé, glissade, pas de chat, battement devant to 90 degrees, développé devant to 90 degrees.
Stage 4	All steps in stage 3 plus: fouetté turns, a la seconde turns, float turns, rond de jambe en l’air, battement devant to full range, développé devant to full range.
Stage 5	All steps in stage 4 plus: cambré to the front, soubresaut, changement, sissonne, assemblé, temps levé.
Stage 6	All steps in stage 5 plus: saut de chat, grande jeté, tour jeté. Return to partner work. Pointe work can be started after pain-free completion of this stage.

Neck Injury Return to Ballet Protocol	
Stage 1	All steps should be completed with barre support first progressing from two-handed support to one-handed support. Consider using a scarf around the neck during warm-up. All barre, no restriction except no cambré (i.e., no turns, no jumps).
Stage 2	All steps in stage 1 plus: cambré side and front, detourné.
Stage 3	All steps in stage 2 plus: cambré back, soubresaut, changement, sissonne, assemblé, temps levé.
Stage 4	All steps in stage 3 plus: saut de chat, grande jeté, tour jeté.
Stage 5	All steps in stage 4 plus: all turns: start with single turns and progress when pain-free (pirouettes, fouetté turns, a la seconde turns, float turns, attitude turns, piqué turns, chainé turns).
Stage 6	All steps in stage 5 plus: return to partner work.

Our protocol uses the language of ballet; however, we believe it could potentially be extrapolated to be used for modern, contemporary, and jazz dancers. This dance-specific, late-stage rehabilitation protocol was developed based on review of the general sports medical literature, review of expert opinion, and the lead author’s (physician, LG) and second author’s (physical therapist, MF) extensive clinical experiences in dance injury practices.

A glossary of common ballet terms is included in Table 2 [14].

**Table 2:** Ballet Term Glossary MTP: metatarsophalangeal [14].

Term	Definition
A la seconde turn	A turn where the working leg is abducted to 90 degrees while remaining turned out. The supporting leg pliés between turns.
Allégro	Fast steps and jumping movements in center. Petite allégro are smaller jumps such as petit assembles and jetés, medium allégro includes sissones and entrechat cotes and grande allégro typically includes grande jetés, cabrioles, fouettés en l’air, saut de chats.
Arabesque	Standing on one leg with the other leg extended back in the air with a straight knee.
Assemblé	A jump from one foot landing on two feet.
Attitude	Movement including extension or flexion at hip with knee flexion.
Cambré	To bend at the waist either forward or backward.
Chainé turns	“To chain”. It is a traveling turn with quick, connected small steps alternating between feet. Connected chainé turns are “chained” together.
Changement	“To change”. It is a jump using both feet where the dancer begins in 5^th^ position in plié, jumps up with straight legs, switches legs and ends in 5^th^ position with the opposite foot in front.
Chassé	One foot gliding forward leading with the toes, the second leg then quickly shoots in to meet it.
Degagé	“To disengage”. This involves extending the leg while pointing the foot slightly off the floor and then more forcefully bringing the leg back in.
Demi	“Small”.
Derriére	“To the back”
Devant	“To the front”.
Développé	“To unfold”. Large lower extremity movement where working leg is moved into knee flexion to supporting leg and then into extension in the open position in the air.
Echappé	“To escape”. The feet slide from one position, usually fifth, out to another position (second or fourth) then back to the starting position.
En l’air	“In the air”
En tourant	“To turn”
Float turns	Turns with the leg flexed at the hip and extended at the knee in second position. Turns which are performed without lowering to plié between turns.
Fondu	“To melt”. Demi plié on one leg.
Fouetté	A pirouette (turn) performed with a circular whipping movement of the gesture leg extending and then flexing with the foot returning to touch the supporting knee.
Frappé	“To strike”. The working leg is flexed in front of the ankle, shoots out to strike the floor, then comes back in flexed behind the ankle.
Glissade	“To glide”. It is a traveling, usually small, jump that is used to link other steps together.
Grande	“Large”
Grande battement	A kick or lift of the leg in the air flexed at hip and extended at knee.
Jeté	“To throw”. It is a jump from one foot to the other with the working leg moving through battement.
Pas de bourrée	Three small steps alternating the feet moving back, to the side, then to the front.
Pas de chat	“Cat’s step”. A jump from one foot to the other where each leg moves though passé. There is a moment in the air where both legs are in high passés with pointed feet. There are variations in 4^th^ and 5^th^ positions.
Passé	“To pass through” from one position to another.
Penché	“Leaning”. It is a position with the working leg in arabesque and the torso leaning forward. The height of the arabesque is often idealized so that the standing leg and working leg are at 180 degrees.
Piqué turns	“Pricking”. It describes the entry into the turn where the dancer steps directly into full pointe or high demi pointe as they begin the turn with an extended knee. The working/gesture leg may be in any position including arabesque, attitude, etc.
Pirouette	A turn on one foot with the raised foot of the gesture leg touching the knee of the support leg.
Plié	A bend at the knees and ankles.
Port de bras	“Movement of the arms”. This describes how a dancer moves their arms from one position to another.
Dance Positions	All positions are performed in turnout with fully extended knees.1^st^ position is standing with the heels together, 2^nd ^position is standing with the feet next to each other but apart, 3^rd ^position is standing with the heel of one foot next to the arch of the other foot, 4^th ^position is standing with one foot in front of the other but apart, and 5^th ^position is standing with the heel of one foot next to the toes of the other foot.
Relevé	“To rise”. The dancer’s weight is on the balls of the feet at the MTP joint or on the toes if the dancer is in appropriate shoe wear.
Rond de jambe	Circular movement of the leg.
Saut de chat	“Cat’s jump”. A jump from one foot to the other with the leading leg moving through développé and at the height of the jump in air the dancer is in the split position.
Sauté	A jump from two feet landing on two feet.
Sissonne	A jump from two feet landing on one foot.
Soubresaut	A jump from two feet landing in 5^th^ position.
Support leg	Weightbearing leg
Temps levé	A jump from one foot landing on the same foot.
Tendu	Leg extension with a pointed foot where the foot remains in contact with the ground.
Tour jeté	A grande jeté performed while turning so the dancer lands the jump in arabesque facing the direction they came from.
Turn out	Ballet technique where hips are externally rotated.
Working leg	Non-weight-bearing leg, also referred to as the gesture leg.

The Fundamental Phases of Healing

An understanding of the basic science of tissue healing and rehabilitation is required to explain the importance of a proper progressive, late-stage rehabilitation RTS program in ballet. To summarize, the three phases of tissue healing are: inflammation, repair (proliferative), and maturation (remodeling) [3,16].

The inflammation phase lasts approximately 5-14 days and is characterized by bleeding, hemostasis, and platelet granulation. This phase initiates the healing process by attracting local stem cells to the area and is distinguished by pain, edema, warmth, and dysfunction. Extremes of inflammation, insufficient or excessive, can result in inadequate or incomplete healing [3,16].

The repair (proliferative) phase is the second phase of tissue healing. While the timing of all the phases overlaps to some extent, the second phase of healing lasts for weeks to months and is defined by neovascularization, the formation of type three (immature) collagen, and the formation of fibroblasts and growth factors which promote cellular healing. Although pain resolves during this phase, re-injury is common because the healing tissue does not have the same tensile strength as mature tissue [3,16].

The maturation (remodeling) phase, or the third phase of healing, involves the conversion of type three (immature) collagen, to type one (mature) collagen and can occur from weeks after an injury to one year. This phase also results in collagen fiber realignment in response to load, emphasizing the importance of appropriate, progressive loading in late rehabilitation. The maturation (remodeling) phase of tissue healing is also associated with reduced cellularity and vascularity, cross-linking of the mature collagen fibers, and scar tissue maturation. Tissue healing continues well past the resolution of the patient’s symptoms; therefore, proper rehabilitation and correction of underlying form defects as well as strength and neuromuscular control imbalances are critical in preventing re-injury [3,16].

It is further beneficial to consider the approach to the rehabilitation of a ballet dancer in three stages: the acute phase, the recovery phase, and the functional RTS phase, also referred to as late-stage rehabilitation [4]. The stages of rehabilitation correlate with the stages of tissue healing described above.

The acute phase of rehabilitation parallels the inflammatory phase of healing; therefore, pain and edema control are the primary goals during this phase. This is the shortest phase of the rehabilitation pathway [6,8,22-24].

The recovery phase of ballet injury rehabilitation involves the restoration of fitness characteristics such as motion, strength, and endurance. This phase can be thought of as basic rehabilitation. It parallels the repair (proliferative) phase as well as components of the maturation (remodeling) phase of tissue healing. Goals during this phase of rehabilitation not only include restoring fitness parameters mentioned above but also treating neuromuscular and biomechanical deficits in motor control that influence performance [6,8,22-24].

Finally, the third and final phase of rehabilitation is the functional phase, also known as late-stage rehabilitation. Late-stage rehabilitation immediately follows the completion of basic rehabilitation. The primary goal during this phase is to prepare the athlete for RTS. This phase corresponds to the later part of tissue remodeling and is also known as the maturation phase of healing. The rehabilitation goals during this functional phase include controlled, progressive increases in sports-specific demands [2,9,25]. Dance medicine professionals need validated late-stage rehabilitation protocols with controlled, progressive increases in ballet-specific demands to assist ballet dancers in safely returning to their sport during the functional phase of rehabilitation after completion of the recovery phase of rehabilitation [4].

## Review

Method of literature search

Search Strategy

**Figure 1:**
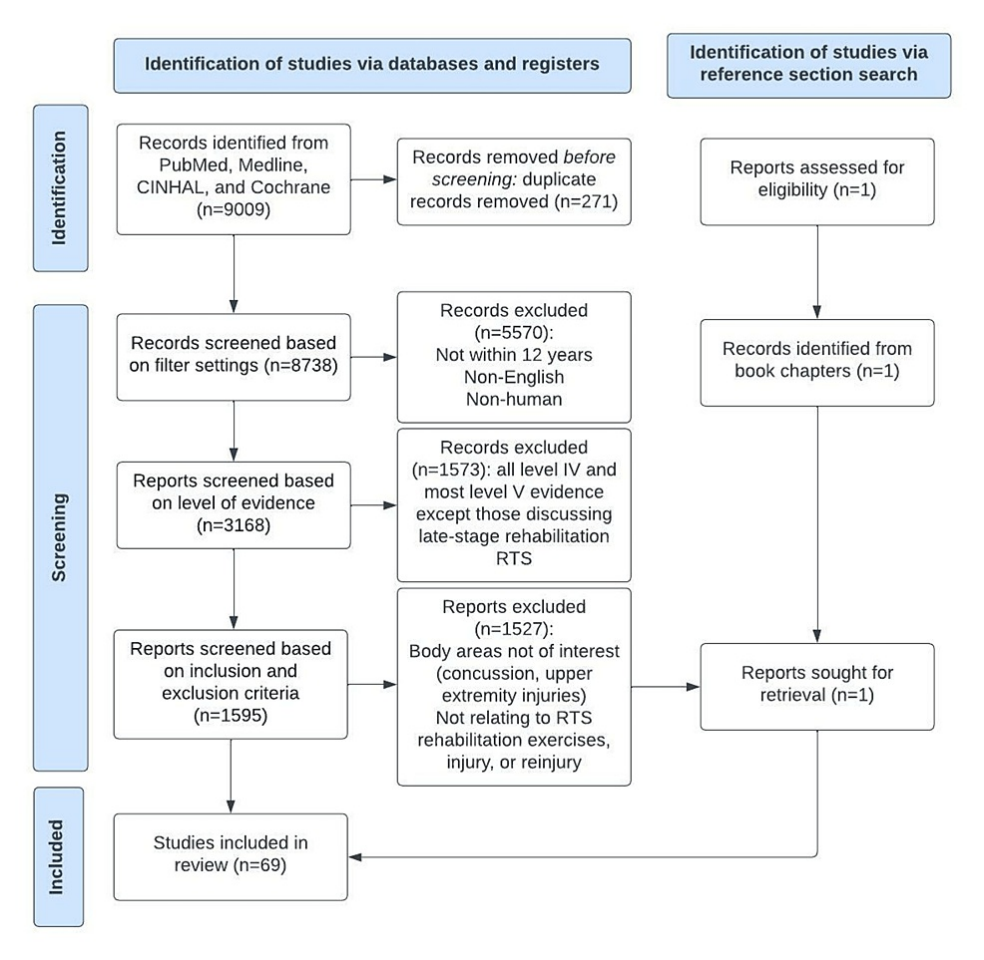
Exclusionary Cascade of Articles Reviewed RTS: return to sport

A broad database search of MedLine, PubMed, CINHAL, and Cochrane Library was performed on June 22, 2022, using keyword combinations to search the literature for key aspects relevant to our area of interest. The following search terms were entered for our review: “dance injur*” or “ballet injur*”; “return to sport” or “return to play”; “dance rehabilitation”; and “reinjur* in sport,” to identify suitable articles for this review. The complete search strategy is presented in Figure 1.

Evidence Acquisition

Our database search (MedLine, PubMed, CINHAL, and Cochrane Library) yielded a total of 9009 records. Additionally, review articles were scanned, and the bibliographies of all articles were thoroughly examined for additional studies pertinent to the topic of the review. We removed 271 duplicates. We excluded 5570 articles not published in English, and all articles based on non-human subjects. A filter was applied for publication dates within the last 12 years. The remaining 3168 articles were divided equally between eight reviewers who worked independently to collate data based on study type and reported conclusions. The Oxford Centre for Evidence-Based Medicine Levels of Evidence (LOE) tool was used to assess the level of evidence in studies.

Evidence Synthesis

For ballet injury, re-injury, recovery, and generalized timing guidelines for RTS, we prioritized clinical trials (randomized and non-randomized control trials), cohort and cross-sectional designs, meta-analyses, and systematic reviews. The studies were assessed for clear study designs with unambiguous inclusion and exclusion criteria as well as for information about ballet injury, criteria affecting re-injury in sport, readiness to RTS, and any specific late rehabilitation protocols. The reference sections suggested one book chapter that was not identified in the primary search and was included. We excluded 1573 low-level studies including all level IV evidence (case series and case reports).

Though our search revealed generalized evidence-based studies informing our discussion of ballet injury, re-injury, recovery, and the initiation of sports-specific movements (RTS), the search failed to uncover evidence-based studies with ballet-and-body region-specific RTS rehabilitation protocols. Therefore, the remaining 1595 records were evaluated by three reviewers (LG, GF and BV) to determine final inclusion based upon the inclusion of re-injury factors, any relevant late rehabilitation (RTS) or movement re-training methods, and body areas of interest (e.g., spine and lower extremity injuries). Articles that pertained to body areas not of interest (e.g., concussion, wrist, elbow, shoulder, hand) and articles not relating to RTS, rehabilitation methods, injury, or re-injury were excluded. Studies were also excluded if they only focused on the immediate post-surgery period and did not discuss the subsequent later rehabilitation with a sports-specific ramp-up. The investigators discussed the articles while considering the inclusion and exclusion criteria until 100% agreement was achieved. In seeking high-level articles relating to ballet injuries and when to start sports-specific movement, level of evidence and scope of inquiry were key components when determining final inclusion. Any conflicts were resolved with discussion. As such, we included 18 Level V studies (expert opinions, narrative reviews, consensus statements, and book chapters) referencing RTS rehabilitation protocols to aid in the development of our novel “Return to Ballet” protocol. This review identified 69 articles that met the pre-specified search criteria. There were three RCTs, 22 systematic reviews/meta-analyses, 15 cohort studies (11 prospective, four retrospective), 11 cross-sectional studies, 15 narrative reviews/consensus statements, and three book chapters (Figure 1).

The need for a ballet-specific return to sport protocol

Being both a sport and a performance art, ballet is highly physical, technically demanding, and has unique rehabilitation requirements [26]. It is reported that 95% of ballet dancers are injured during their careers, with an injury rate of 0.6-4.6 per 1000 exposure hours, which for many ballet dancers, correlates to 25-50 weeks of participation [27-32]. This wide range of injury rates reflects not only novice to professional status, but also differences in how injury is defined, emphasizing the need for reporting standardization [17,33]. Seventy-five percent of ballet injuries are overuse versus acute trauma and most often, involve the lower extremities, especially the foot and ankle [33,34]. The second most common group of injuries in ballet involves the spine, with a high rate of thoracic and lumbar type pain [21,26,35]. Table 3 summarizes common dance injuries and technical considerations in returning to sport.

**Table 3:** Common Ballet Injuries and Ballet Specific Considerations FHL: flexor hallucis longus; MTP: metatarsophalangeal; MT: metatarsal; RED-S: relative energy deficiency in sport; ITB: iliotibial band; DJD: degenerative joint disease; ACL: anterior cruciate ligament; TFL: tensor fascia lata [1,14,15,17,24,28,30-32,36-44].

Body part	Injury	Dance-specific considerations
Foot and ankle	FHL tenosynovitis	The FHL becomes compressed in the proximal margin of the fibro-osseous tunnel along the posterior medial talus under the sustentaculum talus. Pointe shoes change the anatomic positioning and cause the ankle invertor and evertors to be plantar flexors as well as dynamic stabilizers of the ankle.
	Bunions, MTP capsulitis, hallux valgus	On pointe bone forces on the MTP joint are up to 12 times the dancer’s body weight (these forces are greater with errors in technique). Weight on the great toe is estimated at 20 kg/cm.
	Midtarsal joint sprain	A common correctable misconception of many ballet dancers is the belief that forcing the forefoot into greater equines by bending the supporting knee with the weight of the body on the dorsum of the opposite foot at barre improves pointe.
	Ankle sprain	Injury risk increases with too much pronation. Restricted posterior glide of the talocrural joint can continue past the point where dorsiflexion appears to have been restored after an ankle sprain. There is an increased risk for future ankle sprains in the ipsilateral and contralateral ankle.
	Achilles tendonitis	Injury risk increases with too little ankle dorsiflexion, hard landings, and excessive pronation on landing.
	Anterior ankle impingement	The ballet dancer will usually report a feeling of being “stuck” with demi plié and painful dorsiflexion from either soft tissue inflammation or tibio-talal osteophyte formation. Treatment of this injury may involve surgery.
	Posterior ankle impingement	The ballet dancer will usually report pain with forceful or repeated plantar flexion, especially on pointe. Check for os trigonum. Treatment of this injury may require excision.
	Stress fracture of foot and lower leg	Most commonly involves proximal 2^nd^ and 3^rd^ MT, base of 5^th^ MT, navicular, sesamoid, midshaft tibia, and distal fibula. Assess control of landing and floor type. Ensure proper warm-up alignment, foot care, and assess for RED-s.
	Strain of medial head of gastrocnemius at the musculotendinous junction	Assess for a proper dynamic warm-up and strengthening once healed.
	Plantar fasciitis	A common injury resulting from forced turnout. Check for irregular landing mechanics especially pronation on landing.
	Peripheral nerve compression	Most commonly includes dorsal cutaneous compression, sural neuritis, and Morton’s neuroma. Check the fit of pointe shoes.
	Onycholysis Paronychia	Protect nail with polish or taping. Avoid removing toenail as nails are critical to a ballet dancer. The preferred treatment is soaking, elevation, and antibiotics. Check the fit of pointe shoes, change the brand of shoes if necessary, and assess the floor surface.
Hip	Piriformis/obturator internus strain	Ballet dancers often overuse hip external rotators when hip abductors are not engaged in stability.
	Snapping hip, hip flexor tendonitis, bony avulsions, sub-spine impingement, sartorius enthesopathy, hip labral tears, hip DJD	Tight ITB can limit the range of motion of adduction and stretching may be indicated. Many ballet dancers are weakest at the end ranges of motion and “throw” themselves into large end range leg flexion movement with uncontrolled hip hiking in positions such as grande battement and développé instead of controlling motion through the end range.
	Stress fracture femoral neck	Assess for RED-s, nutrition, smoking, and alcohol history.
Knee	Patellofemoral syndrome	Assess for forced turnout, a common compensation pattern for less native turnout. Instead of using their deep hip external rotators to achieve an ideal “top down” turnout, dancers compensate by overpronation of the feet at the subtalar joint, external tibial torsion, valgus stress at the knee, quad gripping, increased hip flexion, and increased lumbar lordosis. Often, the ballet dancer assumes this position while their knees are bent in plié and then straightens their knees with fixed feet using the friction of the floor. This improper and potentially injury-inducing movement pattern is referred to as “bottom up” turnout. A dancer should be able to slide from parallel into first position without lifting their feet off the floor to avoid “forced turnout”. The dancer can be instructed to use turnout discs to train their deep hip external rotators. Assess the dynamic position of the knee during landing/squatting and correct valgus or transversus abdominus activation issues, hip flexor tightness, poor hip abductor and/or hip adductor recruitment.
	Patellar tendonitis/osis Osgood Schlatter Patellofemoral Syndrome	Address the ballet dancer’s landing mechanics. Correct high vertical and braking ground reaction forces during landing.
	Quad tendonitis/osis	Ensure the ballet dancer is achieving alignment over the pointe shoe toe-box. Check for overly worn/“dead” pointe shoes which collapse and don’t support the dancer.
	ACL injury	The low rate of non-contact ACL injuries in ballet can be explained by the turnout position which requires greater gluteus activation and decreased knee adduction as compared to many other sports. However, in the case of ACL reconstruction surgery, if the ballet dancer suffers even minimal loss of terminal knee extension, it is often career-ending.
	Meniscal tear	Ballet dancers can have meniscal tears with a rotational force on the knee. Meniscal repair is the most common surgical procedure related to a dance injury.
	Posterior capsulitis and stretching	Injury can occur with excessive extension of the knee, so lax joints must be protected.
Lumbar Spine	Lumbar strain/pain/spondylolysis	The most common dysfunctional movement pattern associated with back pain is increased force across the lumbar spine from an inability to extend the hip without spinal compensation. Back pain can be associated with decreased thoracic, shoulder and/or hip mobility and is often seen in arabesque positions where the dancer is over firing lumbar muscles instead of properly using the posterior chain (e.g., the gluteus maximus and hamstrings). During growth spurts, hyper-lordosis can occur from the tight lumbodorsal fascia. Assess and correct the dancer for a “hinge point” at the lumbar spine so that lumbar extension forces are evenly dispersed across the shoulder, thoracic spine, lumbar spine, and hips. Ensure that transversus abdominus activation is present throughout the movement. Check for forced turnout as this aberrant pattern is associated with lumbar compensation. The dancer should achieve turnout by firing the deep hip external rotators, while exhibiting a neutral lumbo-pelvic alignment and avoiding over-tucking and gripping of quads and gluteus. The dancer should avoid excessive knee hyperextension and foot pronation with turnout. For sacroiliac back pain, any tightness in hip flexors, piriformis, hamstrings, TFL and ITB should be addressed.

There are literature-based generalized RTS guidelines attempting to direct when athletes can begin sports-specific skills. For example, Serner et al. (2020) concluded that soccer players who successfully completed criteria-based exercise testing to define the end of their basic rehabilitation (recovery) phase prior to starting the functional (RTS/late rehabilitation) phase, had a significantly lower re-injury rate than athletes who did not [25]. However, the available protocols directing when to start sports-specific movements are not specific to ballet [5-10,18,23,25,26,45-50]. For ballet dancers returning from injury, there is a need for validated criteria to determine when a ballet dancer is ready to begin RTS and protocols to safely direct the dancer to full participation.

The few RTS dance protocols that have been reported in the literature do not include specific body regions or ballet-specific skills and focus more on generalized principles of balance, jumping and turning, with instructions to progress from barre work, to center, to small jumps, to large jumps, to pointe, and then to partner work [14,15,17]. While this type of generalized approach can be helpful, it fails to address the nuances of how injury to a specific body region may affect the dancer's ability to correctly and safely execute certain ballet skills. Likewise, these generalized RTS principles lack insight into how a dance medicine clinician should subsequently begin to incorporate and progress certain ballet-specific movements depending on the injured body region. Our "Return to Ballet Protocol" is unique in that it expands upon the aforementioned generic dance progression in its careful consideration for both ballet- and body region-specific movements in progressing RTS protocol. For example, generic return to dance protocols advise ballet dancers to resume barre work as the initial step in returning to sport during late rehabilitation. However, a frappé, a step almost exclusively done at the barre, would be an inappropriate and potentially dangerous initial sports-specific skill for a dancer returning to sport following a foot injury. For this reason, our Foot and Ankle Return to Ballet Protocol (Table 1) advises that the dancer resume most barre work but should avoid striking the injured foot against the floor by performing frappé on relevé in stage 2 before safely progressing to a non-modified frappé in stage 3.

The high re-injury rate seen in ballet can be explained, in part, by a lack of sports-specific direction during this vulnerable period of healing following the resolution of pain but before the tensile strength of the injured, immature connective tissue returns to pre-injury levels [6,8,23,25,46,51]. Our “Return to Ballet Protocol” uses a systematic, loading rehabilitation processes in an ordered, ballet-specific sequence of skills to assist medical providers in making complex clinical decisions associated with ramping up ballet dancers back to full participation during the functional (late-stage rehabilitation) RTS phase [23]. No evidence-based study thus far has addressed a late-stage rehabilitation protocol during this critical, but often neglected, phase of rehabilitation. A ballet-specific RTS protocol allows the dancer to prepare for the sport's specific demands in order to maximize safe re-entry into ballet and to decrease re-injury rates [6,51]. As our literature review did not uncover any body region-specific, validated return to ballet protocols, the progressive stages of our protocol were created based on the review of the general sports medicine literature, knowledge of the biomechanical principles of ballet skills, review of expert opinions, and our extensive clinical experience [5,8-10,14,15,25].

Factors contributing to ballet injuries

A goal for a sports medicine provider is to identify the factors impacting recovery from dance injuries. As injury risk is multifactorial, there are multiple strategies that may serve to reduce injury rates and re-injury risk [29,36,37,39]. As many dancers begin their training at a very young age, their early specialization is often associated with no off-season for physical and mental rest [52]. It is common for ballet dancers to increase training hours too quickly as they advance in training, often up to 20-40 hours per week, not including performances. Fatigue, burnout, and technique errors often result [52,53]. This high training load usually coincides with developmental changes associated with growth and puberty, further increasing injury risk [10]. If dance culture were reformed to teach ballet dancers to heed their internal warning signs and to avoid excessive training and burnout, the overuse injury rate would most likely decrease [29,35,38,40,54]. Ekegren’s systematic review reports a study where a high proportion of dancers sustained injuries before age 18 that ultimately ended their young careers [28].

Ballet can sometimes demand biomechanically disadvantageous body positions that may result in injury and motor system dysfunction. A dancer's attempt to achieve a specific "shape" or aesthetic ideal for choreography can push them past their anatomical limitations [33]. Lower extremity injury rates decrease when dancers are screened for femoral anteversion, genu varum and valgum, tibial torsion, and pes cavus and are then trained to work within their individual limitations [17,24,35,53]. As the risk of a lower extremity injury among dancers is substantially increased by a lack of ankle dorsiflexion, improving ankle dorsiflexion decreases injury [55]. There are multiple studies in the dance science literature that associate increased injury risk in dancers with lower muscle strength [26,56], but a systematic review by Moita et al. (2017) argues against this, concluding that improved muscle strength in dancers was not protective against injury. Conclusions reached by Moita are muddied by the fact that most dance studies included in the review did not clearly define injury and included incomplete and non-dance-specific measurements for muscular endurance and strength [41].

Injury risk decreases when a ballet dancer refrains from alcohol and cigarette use [26,53]. Practices and performances on even, sprung (suspended) floor types result in lower forces on the dancers’ extremities and therefore, fewer injuries [32,53]. Injuries are also reduced when pointe shoes are replaced before they structurally deteriorate and lose the ability to provide ankle and foot support [24,37].

One of the leading risk factors for ballet-related injuries and re-injuries is improper execution of turnout, a defining dance position. Technically correct turnout is achieved primarily through hip external rotation which then directs lower extremity position at the knee, ankle, and foot [42]. Forced turnout is a compensatory movement pattern specific to dancers, especially in ballet, where dancers with anteverted hips, or tight hip flexors, attempt to increase the degree of foot external rotation by using the friction of the floor against their feet instead of using their deep hip external rotators. The result of this aberrant movement pattern includes torsional strains and various overuse injuries such as patellofemoral pain and lower leg and foot tendonitis [24]. Educating ballet dancers to activate their deep hip external rotators and to stay within their anatomic limitations is important in preventing turnout overuse injuries [36]. “Rotation disks” can be useful in this situation because they teach the dancers the correct technique of activating their hip rotators in turnout [14,17,24].

Given that up to 58% of ballet dancers born female have hypermobility syndromes, special precautions should be taken in this group of dancers to reduce injury rates to their slower healing, more vulnerable connective tissue [14,24,56]. The hypermobile athlete does not benefit from standard flexibility work. Uncontrolled, extreme range of motion coupled with high forces and frequent repetition is not beneficial for any athlete and is especially damaging for a hypermobile dancer. When a hypermobile ballet dancer extends past their anatomic limitations, the joint capsule is stretched rather than the muscle-tendon unit, and the protective neural response to the resulting microtrauma can paradoxically reduce mobility [14,15,57]. At extreme ranges of motion, the hypermobile athlete’s joints often sublux and lead to joint instability, shoulder and hip labral tears, and permanent capsular laxity. Using a ballet-specific rehabilitation protocol to ramp up to full participation after injury is even more important for this group of athletes [14,24,58].

Factors contributing to re-injury in ballet

With the rate of re-injury reported at 30% depending on sport and body part, avoidance of re-injury is key for the healing athlete [6,51]. Most subsequent injuries occur within two months of the initial injury [33,59]. Of these re-injuries, up to 14% affect the same area, and up to 75% will affect a different location [25,32,51,59]. Dance medicine is most effective when rehabilitation treatments focus on functional movement patterns and neural adaptations rather than just the focal injury [6,24,32,37]. Patients with the poorest functional outcomes after ballet injury were those who were older, had chronicity associated with their injuries, were fearful of re-injury, or did not complete physical therapy [35,50].

As stated above, 75% of re-injuries affect a different body part, highlighting the role of regional interdependence. The term "regional interdependence" refers to the interaction between body regions and how dysfunction in one location can contribute to dysfunction in other areas. Regional interdependence and the neurological component of a musculoskeletal injury can assist in explaining why injuries at one site can increase the risk of sustaining another injury in a different body part [11,12,32]. It has long been settled in general sports medicine literature that a previous ankle sprain is a risk factor for re-injury to the ipsilateral and to the contralateral ankle [48]. It is also well known that years after the initial injury, and regardless of surgical intervention, traumatic anterior cruciate ligament (ACL) ruptures can result in significantly reduced maximal voluntary activation of the quadriceps. Further, one-third of hamstring injuries recur within the first two weeks of returning to sport as impaired activation (neuromuscular inhibition) and atrophy of the injured muscle often persist after pain has resolved [13,22,24,60]. The rate of re-injury increases when standard therapy fails to address reflexive stability deficits on the uninjured side [5,11,12,22,61]. While no single ballet rehabilitation strategy can be regarded as the gold standard, it is far too common for the ballet dancer’s health care team to prioritize strength, power, and flexibility in the injured body region while ignoring global biomechanics and neuromuscular control [14,61,62].

In ballet, prevention of injury and re-injury is highly dependent on proper scapular kinetics, pelvic and lumbar position, breath control, and the ability to control the extremities around a stable spine [56,62]. Functionally based therapies often use kinesio tape to assist the ballet dancer in optimizing proprioception and stability [36,38,63].

Pain has been reported to increase re-injury risk by altering central nervous system signaling. The altered signal then interferes with both muscle recruitment and lengthening of the musculotendinous unit which results in re-injury. As training while in pain increases the likelihood of re-injury, pain control is therefore an important factor to be considered while managing injuries [30-32,53].

Although many ballet dancers unnecessarily fear “bulking up”, resistance training programs improve performance and are thought to reduce the risk of re-injury [50]. In addition, it is generally accepted that cardiovascular fitness prevents re-injury as fatigue has been shown to adversely affect a dancer's capacity to sustain the neuromuscular control necessary for functionally stable movement [24,64]. While one study concluded that supplemental cardiovascular training to dance did not reduce injury risk, this systematic review was limited by a small sample size, lower-level studies, short duration of supplemental training (eight weeks), and a lack of dance-specific endpoints [43].

Completion of basic rehabilitation

During basic rehabilitation (parallels the recovery phase of rehabilitation), it is essential to address proper jumping and landing mechanics while maintaining conditioning of the non-injured areas. For example, it has been documented that the average ballet dancer may jump up to 200 times in a 90-minute class, demonstrating the importance of proper movement retraining [41,65]. One may observe a ballet dancer performing, what appear to be, high-level activities, such as grande allégro; however, a focused analysis may reveal a lack of controlled movement. Although it may seem that the dancer is correctly executing an expansive jump, the dancer may be using momentum to simply “throw” the limb into the end portion of the range of motion. This can demonstrate a lack of full motor control and a failure to address this inadequacy during injury rehabilitation will likely result in re-injury. Rehabilitation must not only focus on dance fitness but also on identifying and correcting underlying dysfunctional movement patterns [5-7,9,51].

Despite the importance of neuromuscular proprioceptive exercises in most of the general sports literature, Postle’s systematic review of ankle injuries found that with the addition of ankle proprioceptive exercises, there was not a statistically significant difference in the occurrence of recurrent ankle injury [61]. Postle’s systematic review, however, did conclude that neuromuscular rehabilitation improved function and reduced subjective feelings of instability [61].

As stated previously, it has been established in the general sports medicine literature that athletes have a 30% risk of re-injury after returning from injury. One potential explanation for the increased risk of re-injury is the lack of available evidence-based testing for “biologic readiness” of tissue healing, defined as when tissue has returned to its pre-injury tensile strength. Without such a tool, it is imperative that a dance medicine practitioner incorporate a timely, effective RTS protocol upon assessing the movement patterns [6,51].

Because ballet has many unique movements not seen in other sports, it is our opinion that an injured ballet dancer would benefit from an in-person individual assessment from a licensed medical provider with dance experience. A dance medicine professional considers what exercises are possible to continue during recovery from injury, and can assist in providing an individualized plan. In the acute rehabilitation phase, for example, the ballet dancer can often perform dance skill visualization exercises and a non-weight bearing ballet barre series performed lying on the floor or floating in a swimming pool. During the recovery phase, the dance medicine specialist can assist in advancing a medical plan with the goal of maintaining strength and neuromuscular control with relative rest and protection of the injured body part [22,24,61]. Finally, the dance medicine clinician can assist in determining when the ballet dancer is ready for the final phase of rehabilitation, the functional phase, and assist in safely progressing an athlete through this late rehabilitation RTS phase. Our protocol can be of assistance in this endeavor. Unfortunately, a large proportion of general clinicians advise a period of complete rest after an injury and then expect the ballet dancer to be able to RTS with the instructions to “take it easy at first”. Our experience has shown that providing a structured, progressive ballet-specific RTS rehabilitation protocol is a more effective approach to preventing re-injury.

When to begin return to ballet protocol: seven objective criteria for readiness

Our review resulted in the development of seven evidence-based criteria to signify the end of basic rehabilitation and the dancer’s readiness to begin the RTS protocol. These recommendations are intended to serve only as a guide and require that the licensed medical clinician critically evaluate if the athlete is ready to safely begin our “Return to Ballet Protocol” based on injury type and variation in healing.

One: Goals of basic cardiovascular fitness should be demonstrated by a validated method. One such test is the “accelerated 3-minute step test” which measures heart rate recovery after three minutes of stepping on and off a 12-inch-high step to a 112 beats per minute metronome tempo [17,66].

Two: The dancer should demonstrate proper diaphragmatic breath and rib control, control of the transversus abdominus, deep hip external rotators, and foot intrinsics with speed, intensity, and surface instability [14,17,26,38,56].

Three: The dancer should be able to perform a single leg stance for at least 30 seconds [24,44,48].

Four: Motor strength at the hip and knee should demonstrate muscular control through the entire range of their mobility, rather than just relying on momentum to achieve the end range of motion [26,67,68].

Five: Neuromuscular deficits and ballet technique errors should be corrected with a reeducation of dysfunctional movement [22-24,38,61]. This would include the recovery of balance, coordination, and agility with motor control in all planes of movement [6,48].

Six: The ballet dancer should exhibit a pain-free range of motion and near-equal muscle strength (within 80-90%) when comparing the affected extremity to the unaffected extremity [6,8,9,58]. Symmetrical strength has been shown to be more important than maximum strength when returning to all sports, including ballet [5,18].

Seven: As recovery progression and retention of movement patterns are affected by sleep, stress, and nutrition, these factors should also be addressed and corrected prior to starting our “Return to Ballet Protocol” [5,6,10,18,36,69].

Individualizing progression through the return to ballet protocol

If the injured ballet dancer meets the above criteria, they can begin the functional RTS phase with our “Return to Ballet Protocol” (Table 1). Treatment timeframes progressing through the protocol’s stepwise stages may vary and may require adjustment by the medical practitioner based on rates of healing, body part affected, and injury type [10].

Athletes often experience some degree of discomfort in returning to sport and have a difficult time determining when to push through the discomfort or when to stop. As the clinician guides the dancer through the RTS protocol, we advise that the dancer use a modified version of Pearce’s “soreness rules” (Table 4) [9].

**Table 4:** Modified Pearce's Soreness Rules* *Soreness is defined as discomfort greater than 3/10 [9].

Criterion	Action
Soreness during warm-up that continues during practice	Take 2 days off and drop back to previous stage
2-3/10 soreness during warm-up that resolves	Advance as per protocol recommendations
Soreness during warm-up that initially resolves but recurs during practice	Take 2 days off and drop back to previous stage
Soreness that starts the next day and does not resolve in 24 hours	Take 1 day off and resume stage

While progressing through our protocol (Table 1), the dancer’s discomfort should ideally remain a three or below on a scale of 10 when returning to ballet movements [9,46]. Any discomfort should improve with warm-up, and any flare-up of discomfort should subside within 24 hours of the onset [9,10]. When soreness persists, the path through the modified Pearce soreness protocol is recommended [9]. If despite these instructions, the ballet dancer has difficulty progressing to the next phase because of persistent or increasing pain, referral back to a licensed medical clinician should be considered [9,10].

A common initial recommendation for overuse injuries would be for the ballet dancer to remain at each stage for three to five practice days and begin the protocol at 50% of their normal practice volume before attempting to advance to the next stage. Before advancing to stage 2, the athlete should have progressed to 100% of practice intensity for stage 1 (Table 1) [8,10].

It is also important to consider the allowed hours of training as the ballet dancer returns to practice [29]. Depending on the nature and extent of the injury, training hours may need to be limited for several weeks, to even months in some cases.

In the event of multiple trauma, the licensed dance medicine clinician should select a body region-specific protocol based on the most painful injury and progress based on symptoms.

A slower ramp-up time frame is often required for impact-related injuries such as fractures, osteochondral defects, or surgical recovery. For impact injuries, the authors commonly recommend keeping the dancer at each stage of our delineated RTS protocol, as set forth in Table 1, for at least seven practice days and starting at 25% of the normal practice volume before attempting to progress to the next stage. Initially, the ballet dancer should be able to rest between exercises and advance as tolerated. A slower ramp-up will also be required when the dancer has multiple concomitant injuries [8,10]. The ramp-up progression rate for overuse injuries and muscular strains often progresses more quickly.

As the ballet dancer builds strength and dance endurance, less rest between exercises will be required and the hours of practice time will increase. Limiting the number of repetitions of difficult skills to 5-10 per practice is often initially helpful. Learning new skills should not be attempted until the ballet dancer has progressed through the entire protocol [5,6,8,10].

Limitations

One potential drawback of our review is that we found no evidence-based RTS literature on exactly when a ballet dancer would be ready to RTS. Therefore, the objective readiness criteria to initiate an RTS protocol were extrapolated from studies involving other sports: primarily male, college-aged, soccer and American football players. Further, our search revealed no evidence-based, ballet-specific, body region-specific protocols for the functional RTS phase of late rehabilitation. The generalizability of RTS protocols to ballet was often hindered by heterogeneous, small study populations that included sports other than dance. Therefore, we created our protocol based on our clinical experience as well as review of general expert opinion pieces primarily based on other sports. The protocol was developed with an isolated injury in mind. As stated previously, the provider should prioritize the most painful injury when treating a ballet dancer with multiple trauma.

Finally, the limitations of our review of ballet injuries and re-injuries were hindered by the lack of a uniform definition of injury among studies, and heterogenous methodologies in data collection and reporting of injury and re-injury. For instance, some studies defined injuries as any event requiring time away from ballet, but this definition may miss a significant portion of injuries as many ballet dancers continue to perform despite pain.

## Conclusions

The dual purpose of this review was to conduct a comprehensive literature search to identify factors that impact injury, re-injury, and recovery for common ballet injuries, and to provide clinicians with timing guidelines for entering and implementing our novel ballet-and-body region-specific late-stage rehabilitation (functional phase) RTS protocol. Thus far, clinicians have to rely on low-level evidence including informal, incomplete, and heterogeneous expert opinions when recommending when to start and how to progress late-stage RTS rehabilitation protocols. The current body of research regarding RTS in ballet is sparse, replete with bias, and RTS readiness criteria are reported inconsistently and heterogeneously. There is a lack of clear, standardized criteria in the dance science literature to assist medical providers in guiding injured ballet dancers’ safely back to ballet after an injury, especially during the vulnerable phase of tissue healing where pain has resolved but tissue maturation is not yet complete. As ballet has unique movement patterns not seen in any other sports, a consensus in the literature is needed regarding the precise dance moves, balance, and strength requirements required preceding a safe RTS in ballet.

Further research is also needed to fill the existing gaps in the ballet literature to provide clinicians with evidence-based RTS guidance. Our hope is that our “Return to Ballet Protocol” will not only prevent re-injury by directing the ballet dancer safely back into full participation but will also inspire future research by clinicians to standardize ballet-specific RTS decisions. Controlled clinical trials using consistent terminology that compare validated functional performance measures against conventional protocols are needed.
